# Potential Role of Individual and Combined Effects of T-2 Toxin, HT-2 Toxin and Neosolaniol on the Apoptosis of Porcine Leydig Cells

**DOI:** 10.3390/toxins14020145

**Published:** 2022-02-16

**Authors:** Jingru Xu, Zhihui Zhao, Wenbo Guo, Aru Ling, Jianhua Wang, Xichun Wang, Junhua Yang

**Affiliations:** 1 College of Animal Science and Technology, Anhui Agricultural University, Hefei 230036, China; xujingru1231@163.com; 2 Institute for Agri-Food Standards and Testing Technology, Shanghai Academy of Agricultural Sciences, Shanghai 201403, China; zhaozhihui@saas.sh.cn (Z.Z.); guowenbo@saas.sh.cn (W.G.); larjy123@163.com (A.L.); wangjianhua@saas.sh.cn (J.W.)

**Keywords:** apoptosis, HT-2 toxin, individual and combination, neosolaniol, porcine Leydig cells, T-2 toxin

## Abstract

T-2 toxin usually co-occurs with HT-2 toxin and neosolaniol (NEO) in the grains and feed. Our previous studies found that T-2 toxin and its metabolites’ binary or ternary combination exposure to porcine Leydig cells (LCs) displayed synergism in certain range of dosage and cannot be predicted based on individual toxicity. However, the possible mechanism of these mycotoxins’ combined exposure to cell lesions remains unknown. Based on 50% cell viability, the mechanism of apoptosis in porcine Leydig cells was investigated after exposure to T-2, HT-2, NEO individual and binary or ternary combinations. Compared with control, the adenosine triphosphate (ATP) content decreased, reactive oxygen species (ROS) level increased, and mitochondrial membrane potential (MMP) decreased in all treated groups. Additionally, the cell apoptosis rates were significantly increased in test groups (*p* < 0.05), and the B-cell lymphoma 2 (Bcl-2) Associated X (Bax)/Bcl-2 ratio and the expression of caspase 3, caspase 8, cytochrome c (Cytc) in the treated group are all significantly higher than the control group. Moreover, the expression of Cytc and caspase 8 gene in NEO and T-2+NEO groups was significantly higher than that in other individual and combined groups. It can be concluded that the toxicities of T-2, HT-2, and NEO individually and in combination can induce apoptosis related to the oxidative stress and mitochondrial damage, and the synergistic effect between toxins may be greater than a single toxin effect, which is beneficial for assessing the possible risk of the co-occurrences in foodstuffs to human and animal health.

## 1. Introduction

Trichothecenes are agriculturally important mycotoxins that present a potential threat to human and animal health throughout the world, which are a large family of chemically related toxins produced by fungi and could be classified into four types based on the absence or presence of characteristic functional groups [1,2]. Type A trichothecenes are mainly represented by T-2 toxin [3], which has the highest toxicity in all the trichothecenes. An increasing body of studies has demonstrated the presence of T-2 toxin in the food and feedstuffs, especially in cold climate regions or during wet storage conditions. A recent report from the European Union (EU) stated that T-2 toxin was a common contaminant in cereal samples from EU member states, including maize, wheat, and oats [4]. In addition, T-2 toxin is nonvolatile and resistant to degradation in different environments, and therefore animals and humans are at a risk for dietary exposure to T-2 toxin. A great number of studies indicate that T-2 toxin causes a large range of toxic effects in human and animals, such immunotoxicity, neurotoxicity, intestinal or hepatic toxicity, and reproductive toxicity [5,6]. Furthermore, T-2 toxin is associated with an increase in infection rate, activation of oxidative stress, damage of protein and DNA synthesis, and induction of apoptosis and autophagy [6,7,8]. Accumulating evidence has indicated that the existence of T-2 toxin in the feed and food causes immeasurable damage of agriculture and toxic effects in animals and humans. It is important to explore the metabolism of the T-2 toxin in vitro and in vivo, and accurately assess the toxicity of T-2 toxin and its metabolites.

Generally, the major metabolic reactions of T-2 toxin are hydrolysis, hydroxylation, de-epoxidation, and conjugation [9]. Additionally, the most typical metabolic pathway is rapid deacetylation at C-4 and formation of HT-2 toxin [10]. Another important reaction is hydrolysis that produces neosolaniol (NEO), T-2 triol, and T-2 tetraol in mammals. In the natural environment, T-2 toxin and its metabolites frequently co-occur as mycotoxin contaminants in cereals, grains, oat, wheat, and corn byproducts [11,12,13,14]. Therefore, the health risks of T-2 toxin in food are very essential to assess regarding the co-existence of T-2 toxin and its metabolites.

T-2 toxin and its metabolites present a similar toxicity to humans and animals, including inhibition of protein synthesis and cell proliferation, and induction of acute or chronic toxicity [4,15]. However, the toxicity of mycotoxins’ co-existence cannot be predicted based on the individual components, because they may generate the interaction of additivity, synergism, and antagonism [16]. In our previous studies, the combinations of T-2+HT-2, T-2+NEO, and HT-2+NEO on the porcine Leydig cells displayed synergism at low doses but antagonism at high doses, and the ternary combination of T-2+HT-2+NEO revealed an adverse situation from antagonism to synergism [17]. However, the combined toxic mechanism of T-2 and its main metabolites HT-2 and NEO in porcine LCs has been unclear. As a consequence of T-2 toxin treatment, the DNA damage in the LCs is caused by the oxidative stress pathway, and eventually leads to apoptosis and high autophagy and presented a series of toxic effects [18,19,20,21]. In addition, HT-2 and NEO, as metabolites of T-2, usually have similar toxicity characteristics [22,23]. Therefore, the combined effects and possible mechanisms of T-2, HT-2, and NEO induced by single, binary, and ternary combinations were explored with porcine LCs based on our previous study, and to provide ideas and theoretical support for exploring the mechanism of combined mycotoxins on reproductive performance damage in animals.

## 2. Results

### 2.1. ATP Content Analysis

**Figure 1:**
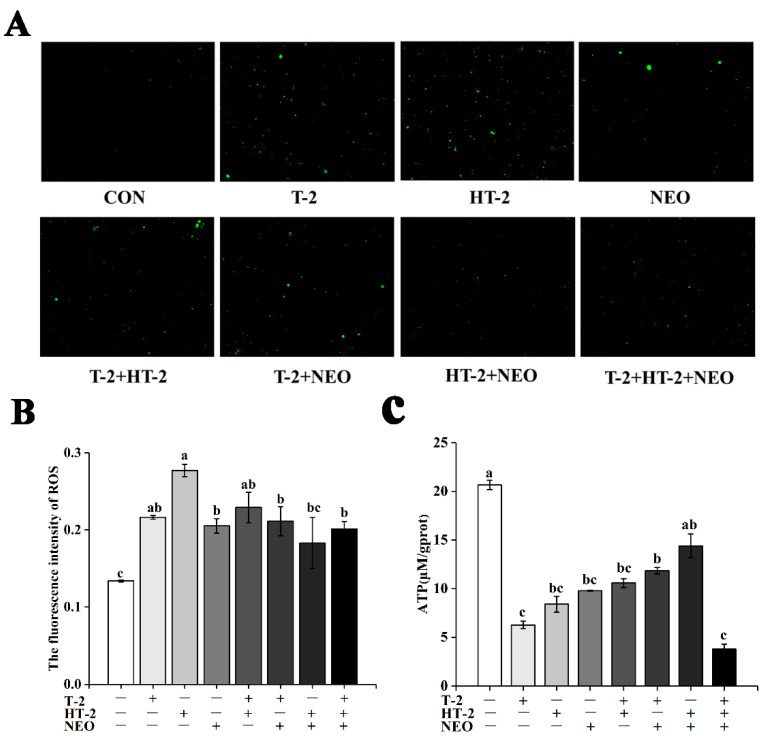
The effects of T-2, HT-2, NEO alone, and binary or ternary combination on content of ROS and ATP in porcine Leydig cells after 24 h exposure. (**A**) ROS levels determined using dichlorodihydrofluorescein (DCFH) fluorescence (magnification, 200×). (**B**) Quantified ROS fluorescence intensity. (**C**) ATP concentration in porcine Leydig cells. Data are presented as mean ± SD from three independent experiments. Values with different superscript letters are significantly different (*p* < 0.05).

Based on individual and combined exposure to T-2 toxin, HT-2 toxin, and NEO, the concentration of ATP was detected and shown in Figure 1C. Compared with the control group, the intracellular ATP content was significantly decreased under 50% cell activity (*p* < 0.05), except that in the HT-2+NEO group. Additionally, the intracellular ATP content in the T-2+HT-2+NEO group was significantly lower than that in T-2+NEO and HT-2+NEO groups (*p* < 0.05), but there was no significant difference between single and binary combined experimental groups.

### 2.2. ROS Level Analysis

Treated with individual and combined exposure by T-2 toxin, HT-2 toxin, and NEO, the level of ROS was determined using the ROS-specific fluorescent dye dichlorodihydrofluorescein diacetate (DCFH-DA) (Figure 1A,B). Under 50% cell activity, the intracellular ROS content in treated groups was significantly higher than that in the control group (*p* < 0.05), except that in the HT-2+NEO group. Additionally, the ROS level in the HT-2 toxin group was obviously higher than that in NEO, T-2+NEO, HT-2+NEO, and T-2+HT-2+NEO groups (*p* < 0.05), and there was no significant difference among other groups.

### 2.3. Mitochondrial Membrane Potential (MMP)

**Figure 2:**
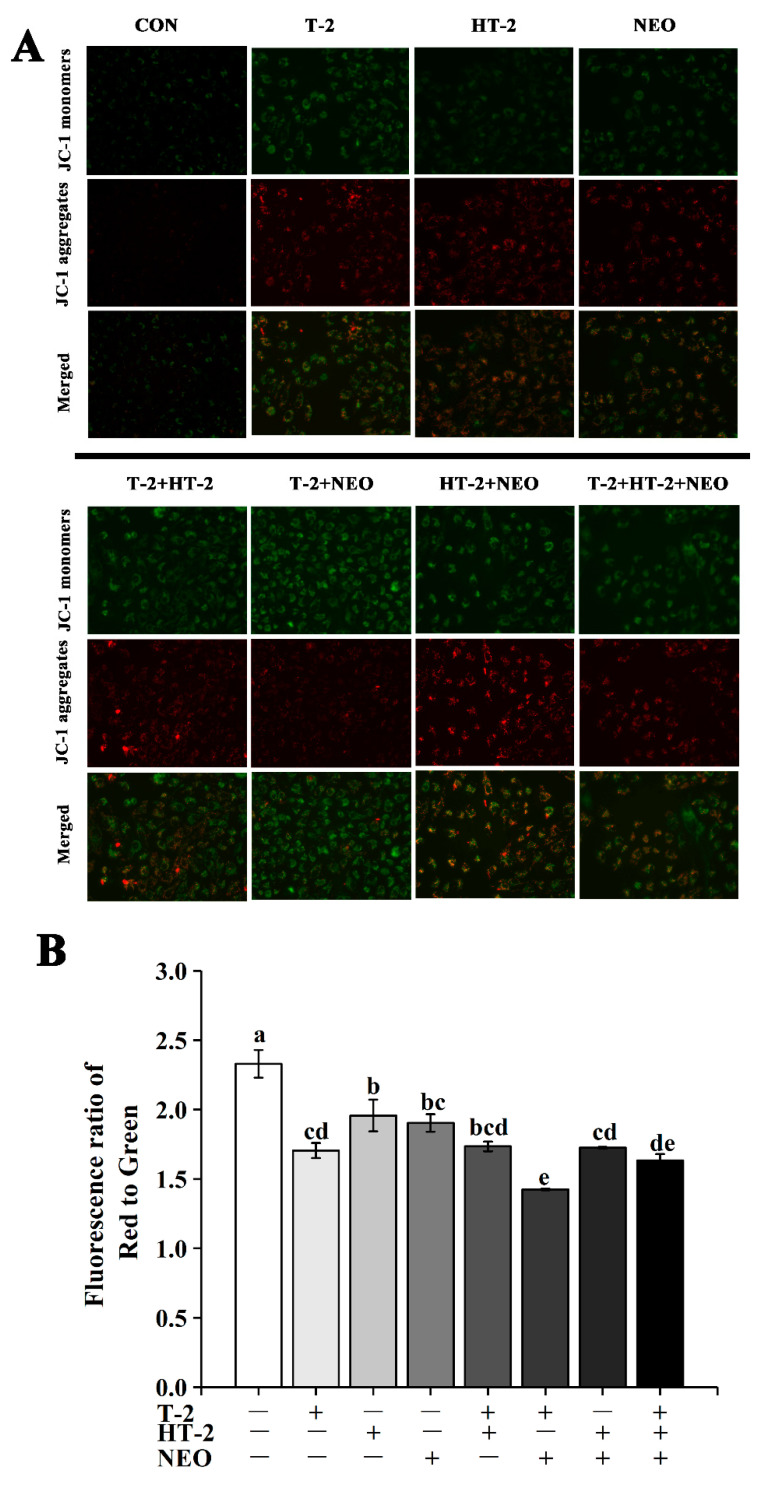
The effects of T-2, HT-2, NEO alone, and binary or ternary combination on mitochondrial membrane potential (MMP) in porcine Leydig cells after 24 h exposure. (**A**) Images of Leydig cells stained by JC-1 and analyzed by fluorescence microscopy (magnification, 400×). (**B**) Quantitative analysis of the ratio of red to green fluorescence intensity. Data are given as mean ± SD (*n* = 3). Values with different superscript letters are significantly different (*p* < 0.05).

The mitochondrial membrane potential (MMP) was evaluated by the 5′,6,6′-tetrachloro-1,1′,3,3′-tetraethylbenzimidazolylcarbocyanineiodide (JC-1) dye. The JC-1 stain in viable cells showed the red fluorescence. Red fluorescence represents JC-1 aggregate and green fluorescence denotes JC-1 monomer, then the merged images showed the combination of green and red images (Figure 2A). The change of mitochondrial membrane potential was indicated by the ratio of red to green fluorescence. Compared with control, the mitochondrial membrane potential of all experimental groups was significantly decreased (*p* < 0.05, shown in Figure 2B). Under 50% cell activity, mitochondrial membrane potential of the T-2+NEO group was significantly lower than the other treatment groups (*p* < 0.05). Additionally, the mitochondrial membrane potential in T-2, HT-2+NEO, and T-2+HT-2+NEO groups was significantly lower than that in the HT-2 group (*p* < 0.05). These results exhibited significant mitochondrial fragmentation after exposure to single, binary, or ternary combinations of T-2 toxin, HT-2 toxin, and NEO.

### 2.4. Apoptosis Assay

**Figure 3:**
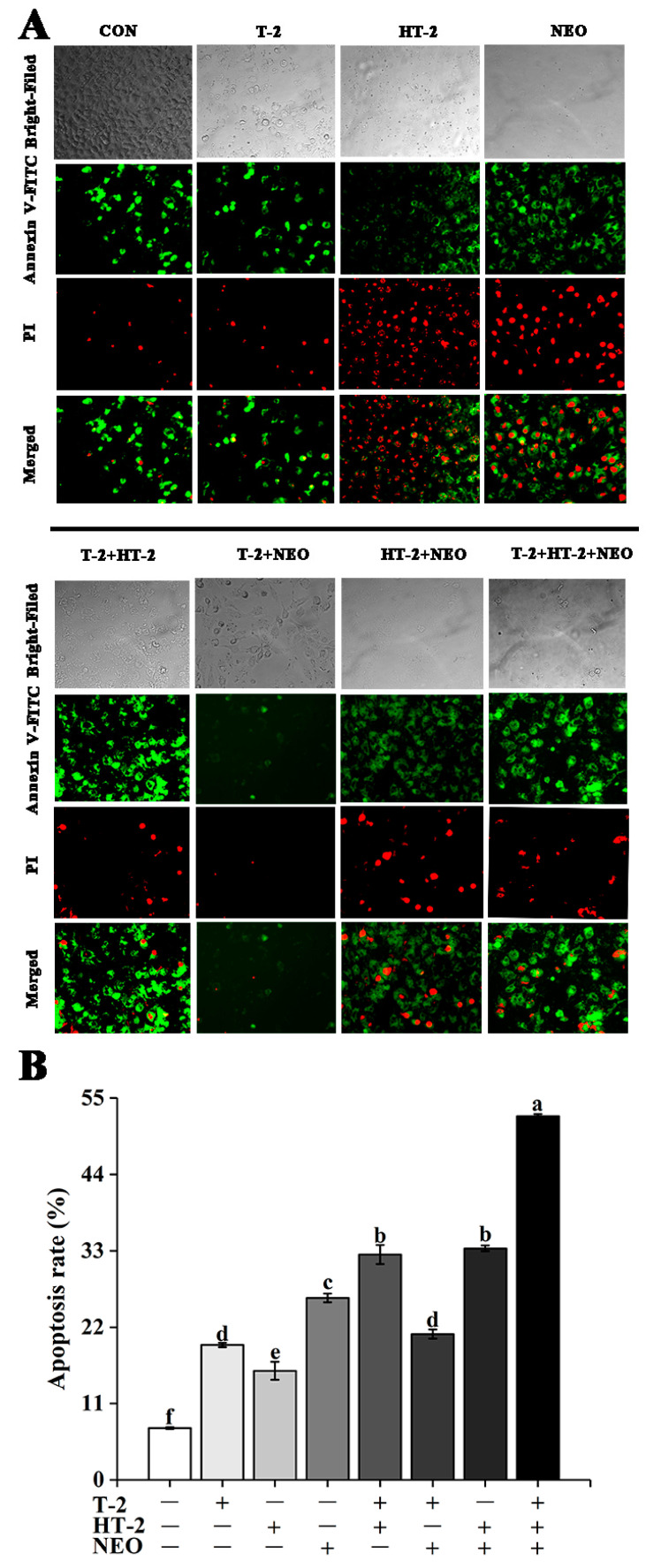
The effects of T-2, HT-2, NEO alone, and binary or ternary combination on apoptosis rates in porcine Leydig cells after 24 h exposure. (**A**) Images of Leydig cells stained by Annexin V-FITC and PI (magnification, 400×). (**B**) Quantitative analysis of apoptosis rate. Apoptotic cells were expressed as percent of total cells counted. Representative fluorescence of 3 independent experiments. Data are given as mean ± SD. Values with different superscript letters are significantly different (*p* < 0.05).

Annexin V-FITC and propidium iodide (PI) staining were rendered. Annexin V-fluorescein isothiocyanate (V-FITC) positive cells were stained with green fluorescence, and propidium iodide positive cells were stained with red fluorescence. Apoptotic cells were stained with only green fluorescence, necrotic cells were stained with both green and red fluorescence, and normal cells were not stained (Figure 3A). As shown in Figure 3B, the apoptosis rate of all experimental groups was significantly higher than that in the control (*p* < 0.05). Under 50% cell activity, the apoptosis rate of the T-2+HT-2+NEO group was significantly higher than that of single and binary combined exposure groups (*p* < 0.05). Additionally, there was no significant difference between the T-2 group and T-2+NEO group, T-2+HT-2 group and HT-2+NEO group.

### 2.5. Expression of Apoptosis-Related Genes

**Figure 4:**
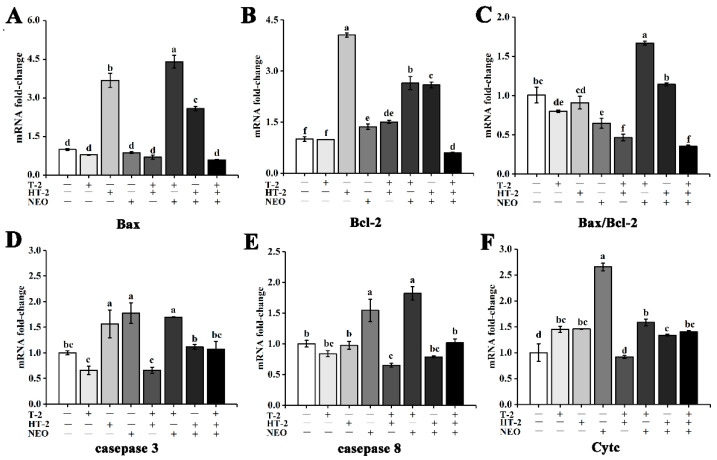
The effects of T-2, HT-2, NEO alone, and binary or ternary combination on expression level of apoptosis-related genes of porcine Leydig cells after 24 h exposure. (**A**) Relative expression of Bax; (**B**) relative expression of Bcl-2; (**C**) relative expression of Bax/Bcl-2; (**D**) relative expression of caspase 3; (**E**) relative expression of caspase 8; (**F**) relative expression of Cytc. Data are given as mean ± SD (*n* = 3). Values with different superscript letters are significantly different (*p* < 0.05).

Compared with control, the expression level of the Bax gene in HT-2, T-2+NEO, and HT-2+ NEO groups was significantly upregulated under 50% cell activity (*p* < 0.05), but there was no significant change in the expression level of the Bax gene in the T-2+HT-2 group and the other groups (shown in Figure 4A). 

Under 50% cell activity, the expression level of the Bcl-2 gene was significantly upregulated (*p* < 0.05), except that in the T-2 group, and the expression level of the Bcl-2 gene was significantly downregulated in the T-2+HT-2+NEO group (*p* < 0.05). Additionally, the expression level of the Bcl-2 gene in the HT-2 group was significantly higher than other groups (*p* < 0.05, shown in Figure 4B).

Compared with control, the ratio of Bax/Bcl-2 in the T-2+NEO group was significantly increased (*p* < 0.05), and the other groups alone and combined were significantly decreased (*p* < 0.05). In addition, the ratio of Bax/Bcl-2 in the T-2+NEO group was significantly higher than that in other single and combined groups (*p* < 0.05, Figure 4C).

Under 50% cell activity, the expression level of caspase 3 in HT-2, NEO, and T-2+NEO groups was significantly upregulated compared with the control (*p* < 0.05). In addition, the expression level of caspase 3 in HT-2, NEO, and T-2+NEO groups was significantly higher than that in T-2, T-2+HT-2, HT-2+NEO, and T-2+HT-2+NEO groups, respectively (*p* < 0.05, shown in Figure 4D).

Compared with the control group, the expression of caspase 8 in the NEO group and T-2+NEO group were also significantly upregulated under 50% cell activity (*p* < 0.05), while T-2+HT-2 exposure induced the downregulation of the caspase 8 level (*p* < 0.05). However, the expression level of caspase 8 in the NEO and T-2+NEO groups was obviously higher than that in the other groups (*p* < 0.05). Moreover, the expression level of caspase 8 in the T-2+HT-2 group was not significantly different from that in T-2 and HT-2+NEO groups, but was significantly lower than other groups (*p* < 0.05, shown in Figure 4E).

Moreover, the expression level of Cytc was significantly upregulated in other single and combined groups (*p* < 0.05). Under 50% cell activity, the expression level of Cytc in the NEO group was significantly higher than other groups (*p* < 0.05), and that in the T-2+HT-2 group was significantly lower than other single and combined groups (*p* < 0.05). In addition, there were significant differences among NEO, T-2+HT-2, T-2+NEO, and HT-2+NEO groups (*p* < 0.05, shown in Figure 4F).

## 3. Discussion

In the present study, we investigated the possible mechanism of T-2 and its main metabolites HT-2 and NEO, individually or in combination inducing the porcine Leydig cells apoptosis. The results showed that under 50 % cell viability, T-2, HT-2, and NEO exposure individually or in combination could induce reproductive cell lesion by triggering the overgeneration of ROS, decreasing the ATP concentration, causing the MMP collapse leading to the disruption of mitochondrial function, and ultimately promoting cellular apoptosis. Additionally, our experiments indicate that the binary and ternary combinations of T-2, HT-2, and NEO displayed synergistic toxicity, and presented a new light to assess the risk of the co-occurrence of the T-2 toxin and its metabolites in food and feed.

Mitochondria, as the main producers in cells, play an important role in maintaining cellular oxidative balance and cell function [24]. ATP is mainly produced by mitochondria and provides energy for biological processes [25]. ROS are also mainly produced in mitochondria and play a dual role in living systems under physiological conditions [26,27]. The stability of mitochondrial membrane potential provides a guarantee for the maintenance of mitochondrial function [28]. Therefore, the changes of ATP, ROS levels, and mitochondrial membrane potential can be used as important indicators to judge the degree of damage to mitochondrial function caused by mycotoxins. In this study, ATP content decreased and ROS content increased in all experimental groups. Similar to previous studies, T-2 toxin can inhibit mitochondrial function through ROS production and ATP content decrease [21,29]. Many reports have shown that T-2 toxin and DON induce apoptosis through a ROS-mediated mitochondrial pathway in different cell types [30,31]. In human primary tubule epithelial cells, T-2 toxin induced apoptosis, whereas its metabolites showed lower cytotoxic effects but still induced apoptosis at higher concentrations [31,32,33]. Maika Königs et al. also proved that T-2 toxin increased the apoptosis rate in porcine renal epithelial cells [34]. In this study, the apoptosis rate of all experimental groups was significantly increased. Similar results found that T-2/HT-2 treatment also significantly increased the number of apoptotic cells and the apoptotic ratio [35]. Other mycotoxin studies have shown that ochratoxin A (OTA) and fumonisin B1 (FB1) presented synergetic cytotoxic effects on rat liver cells (BRL) by inducing apoptosis [36]. These results also explained why the apoptosis rate in the combined group was higher than that in the single group. Our previous study also documented that there is a synergistic effect between T-2 toxin and its metabolites [17]. However, the further research is needed to support this hypothesis.

Both Bax and Bcl-2 are key genes involved in cellular apoptosis. The Bax gene is mainly located in the mitochondrial membrane and promotes the release of apoptotic factors such as Cytc during apoptosis, which then activates caspase 3 to advance apoptosis [21,37]. Conversely, Bcl-2, as one anti-apoptotic protein, could prevent the release of Cytc into the cytoplasm and thus inhibit apoptosis by controlling the permeability of the mitochondrial membrane [38,39]. Therefore, the Bax/Bcl-2 ratio is usually used to assess the occurrence of apoptosis. In this study, the Bax/Bcl-2 ratio and the expression of caspase 3, caspase 8, and Cytc in the T-2+HT-2 group are all significantly higher than the control group. This result is consistent with the change of ROS, ATP, and MMP. Concurrently, the toxic effect in the combined group is greater than in the single group. Our data suggested that a synergistic effect in the combined group was observed, which was consistent with the previous cytotoxic results in our laboratory [17]. Moreover, the expression of the Cytc gene in the NEO group was significantly higher than that in the other single and combined experimental groups, which implies that NEO more easily induces the release of Cytc from mitochondrion and promotes the activation of caspase 3 under the same 50% cell activity [40]. Apoptosis is a caspase-dependent programmed form of cell death, and usually leads to the activation of caspase 8 and then the subsequent caspase 3 [41,42]. Similarity, NEO exposure also obviously promoted the increase in caspase 3 and caspase 8 expression, and it is suggested that NEO may synergistically induce the cellular apoptosis with T-2 and HT-2 toxins. The reason may be that NEO is less toxic than T-2 and HT-2 toxins, and more easily binds with the receptor or metabolizes and converts in the biological processes of cell. However, the potential function of NEO in single or combined groups should be investigated in future research.

## 4. Conclusions

In the present study, we employed porcine Leydig cells to explore the reproductive toxic mechanism of individual and combined T-2 and its metabolites. Based on the 50% cell viability, individual, binary, and ternary combinations of T-2, HT-2, and NEO exposure, the porcine LCs induced the decrease in the ATP content, the overproduction of ROS, the reduction in mitochondrial membrane potential (MMP), and then led to release of cytochrome c from the mitochondria intermembrane space to the cytosol, and finally promoted cellular apoptosis. In addition, the combined exposure treatment presented higher toxicity, which implies that the co-occurrence of T-2 toxin and its metabolites might pose a significant threat to reproductive health.

## 5. Materials and Methods

### 5.1. Chemicals

T-2 toxin, HT-2 toxin, and NEO were purchased from Pribolab Pte. Ltd. (purity > 99%, Singapore). Fetal bovine serum (FBS), dimethyl sulfoxide (DMSO), Dulbecco’s Modified Eagle’s medium (DMEM), Hanks balanced salt solution (HBSS), penicillin–streptomycin (10,000 Units/mL-10,000 μg/mL), 0.25% trypsin/ethylenediaminetetraacetic acid solution (trypsin/EDTA) for cell incubation were provided by Gibco (Thermo Fisher, Shanghai, China). Porcine Leydig cells were obtained from Shanghai Ke Lei Bio-Technology Co., Ltd. (Shanghai, China). All other chemicals, if not stated, were purchased from Sigma-Aldrich (Shanghai) Trading Co., Ltd. (Shanghai, China).

### 5.2. Cell Culture and Treatment

Porcine LCs were inoculated in DMEM medium containing 10% FBS and 1% penicillin–streptomycin, and cultured conventionally in an incubator with saturated humidity of 5% CO_2_ and 37 °C. Porcine LCs were adherent cells and could be subcultured every 2 days.

**Figure A1:**
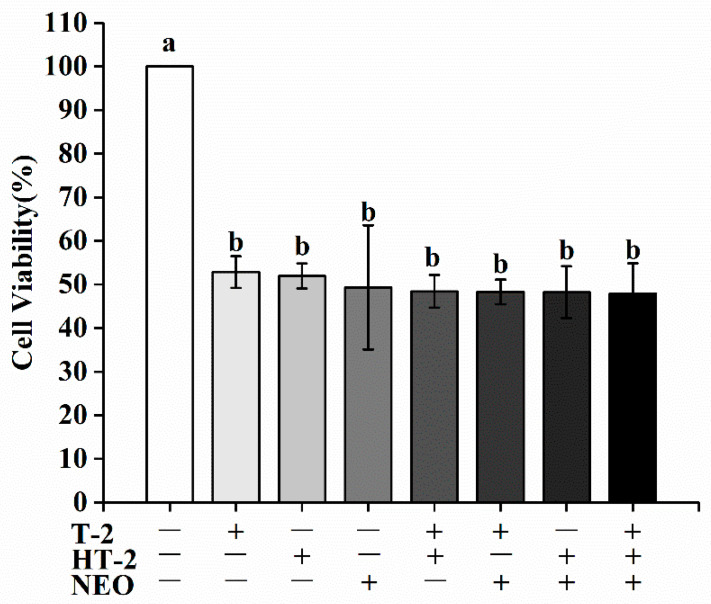
The effects of HT-2, NEO alone, and in binary or ternary combination on expression level of 50% cell viability of porcine Leydig cells after 24 h exposure. Values with different superscript letters are significantly different (*p* < 0.05).

According to the previous dose–effect curve results of T-2, HT-2, and NEO individually and in combination in our laboratory, the doses of the three toxins based on the 50% cell activity were selected to study the apoptotic mechanism [17]. The cells were digested and grafted into a 6-well plate at a concentration of 2 × 10^5^ cells/well. After 24 h, 2 mL complete culture medium was replaced, respectively, by different concentrations of T-2 (0.0125 uM/L), HT-2 (0.025 uM/L), NEO (0.375 uM/L), T-2+HT-2 (0.00625 uM/L + 0.0125 uM/L), T-2+NEO (0.0625 uM/L + 0.1875 uM/L), HT-2+NEO (0.00625 uM/L + 0.09375 uM/L), and T-2+HT-2+NEO (0.003125 uM/L + 0.00625 uM/L + 0.09375 uM/L), while a blank group without toxins was set up. All the doses of mycotoxins were from our previous study [17], and the cell viability is shown in Appendix A Figure A1.

### 5.3. Determination of Intracellular ATP Content

Intracellular ATP levels were determined using ATP assay kits obtained from Beyotime Biological Technology Co., Ltd. (Beyotime, Shanghai, China) [43,44]. After treatment with different concentrations of T-2, HT-2, and NEO individually and in combination, cells were lysed and centrifuged to collect the supernatant. Each well of the blank 96-well plate was incubated with 100 µL ATP detection working solution for 5 min at room temperature. Then, 20 µL of cell lysate and the luminescence were added to each well for assaying immediately by a microplate reader (BioTek Instruments Inc., Winooski, VT, USA). The levels of ATP in each well were calculated according to the standard curve and normalized to the protein concentration of each sample.

### 5.4. Determination of Intracellular ROS Content

Intracellular ROS levels were assessed with DCFH-DA, a ROS indicator that produces fluorescent DCF in the presence of intracellular oxygen, obtained from Beyotime Biological Technology Co., Ltd. (Beyotime, China) [45,46]. Cells were seeded at a density of 2 × 10^5^ cells/well in 6-well culture plates and incubated for 24 h. After exposure with different concentrations of T-2, HT-2, and NEO individually and in combination, cells were washed with Hank’s balanced salt solution (HBSS) and incubated in a 1.5 mL DCFH-DA (20 μM) solution diluted with DMEM at 37 °C for 20 min. Then, the loading solution was replaced with fresh medium. The fluorescence intensity was observed with a fluorescence microscope (Olympus, Tokyo, Japan) and then analyzed by Image-Pro Plus 6.0 software (National Institutes of Health, Bethesda, MD, USA). Three visual fields were selected for each hole to take photos, and three parallel fields were set for each group.

### 5.5. Measurement of Mitochondrial Membrane Potential (MMP)

The MMP was assayed using the fluorescent JC-1 dye purchased from Beyotime Biological Technology Co., Ltd. (Beyotime, China) [47]. The Leydig cells were seeded into 6-well plates with a density of 2 × 10^5^ cells/well and evaluated with different levels of T-2 toxin, HT-2 toxin, and NEO individually and in combination. Both untreated and treated cells were washed with HBSS, and then incubated with 1 mL DMEM with 10% FBS medium and 1 mL JC-1 working solution at 37 °C. After 40 min incubation, the supernatant was aspirated and washed twice with JC-1 buffer, and fluorescent microscopic images of Leydig cells were evaluated using a fluorescence microscope (Nikon, Japan). The degree of mitochondrial damage was assessed by the ratio of the JC-1 aggregates and JC-1 monomers (the ratio of red to green fluorescence). Image-Pro Plus 6.0 software was used to analyze the intensity of red and green fluorescence, respectively. Three visual fields were selected for each hole to take photos, and three parallel fields were set for each group.

### 5.6. Measurement of Cellular Apoptosis 

Cellular apoptosis was performed using an Annexin V-FITC/PI Cell apoptosis assay kit provided by Beyotime Biological Technology Co., Ltd. (Beyotime, China) [48]. In brief, cells were seeded into 6-well plates with a density of 2 × 10^5^ cells/well and evaluated with different levels of T-2 toxin, HT-2 toxin, and NEO individually and in combination. After centrifugation and resuspension in staining buffer, the cells were incubated in Annexin V-FITC and PI solution for 20 min at room temperature. After being observed and photographed under visible light and fluorescence microscopy (40× objective lens), the apoptosis rate was calculated: 

Apoptosis Rate% = (number of apoptotic cells/total number of cells under the same visible light field) × 100%.

Three visual fields were selected for each hole to take photos, and three parallel fields were set for each group.

### 5.7. RNA Extraction and Real-Time Quantitative PCR

Total RNA was extracted from the cells by total RNA extraction kit according to the manufacturer’s instructions. Additionally, the concentration and quality of total RNA was determined by Nano Drop. cDNAs for each RNA sample were prepared using PrimeScript RT Master Mix, then stored at −20 °C.

The mRNA expression levels of Bax, Bcl-2, caspase 3, caspase 8, and Cytc, were quantified using ABI-prism 7500 sequence detection system (Applied Biosystems, Inc., Foster City, CA, USA) and normalized to glyceraldehyde-3-phosphate dehydrogenase (GADPH) reference gene. As shown in Table 1, the primers sequences were designed using Prime Premier 5.0 and synthesized by Sangon Biotech (Shanghai, China) Co., Ltd. (Shanghai, China).

Real-time quantitative PCR reaction was carried out in 20 μL reaction mixtures, containing 10 μL of SYBR Premix Ex Taq (2×), 0.4 μL of ROX Reference Dye (50×), 0.8 μM of each forward and reverse primers, cDNA aliquots, and nuclease-free water. Real-time PCR amplification was performed with an initial denaturation step at 95 °C for 30 s, followed by 40 cycles of 5 s at 95 °C and 31 s at 60~64 °C. The 2^−ΔΔCt^ method was used to determine the relative expression of each gene compared to a reference gene. All samples were amplified in a single PCR run, and each amplification was repeated at least three times [49].

### 5.8. Data Preprocessing and Statistical Analysis

One-way analyses of variance (ANOVA) were performed using SPSS 16.0 for Windows (SPSS Inc., Chicago, IL, USA). The values for mRNA expression levels were presented as fold-change relative to the control group. Significant differences were analyzed by least significant difference (LSD) among different groups at *p* < 0.05 or *p* < 0.01. All data were expressed as mean ± standard deviation (SD).

## Figures and Tables

**Table 1:** Primer sequences and product sizes for qPCR.

Gene	Accession No.	Sequence of Primer Pairs (5′ → 3′)	Product Size/bp
Bax	XM_003355975.2	F: AGCTGAGCGAGTGTCTCAAG	95
		R: AGAAGAGACCACTCCTGGGT	
Bcl-2	XM_003121700.4	F: ACACCTGGATCCAGGATAAC	94
		R: AGAGACAGCCAGGAGAAATC	
caspase 3	NM_214131.1	F: TTGGACTGTGGGATTGAGAC	154
		R: GTGACTGGATGAACCAGGATC	
caspase 8	NM_001031779.2	F: ACTGTCTGGGAGAACAGGAC	147
		R: CCTTAATGTTGTGAAGTCTGG	
Cytc	NM_001129970.1	F: CTGGATTCTCTTACACAGATGC	156
		R: CTATCAAGTCTTCCCTTTCTCC	
GADPH	NM_001206359	F: CACGATGGTGAAGGTCGGAG	180
		R: TTGACTGTGCCGTGGAACTT	

## Data Availability

Data will be provided on request.
